# Randomized clinical trial to evaluate safety and efficacy of convalescent plasma use among hospitalized patients with COVID-19 (PERUCONPLASMA): a structured summary of a study protocol for a randomized controlled trial

**DOI:** 10.1186/s13063-021-05189-6

**Published:** 2021-05-17

**Authors:** Alonso Soto, Fiorella Krapp, Alex Vargas, Lucía Cabrejos, Enrique Argumanis, Patricia L. García, Karina Altamirano, Martín Montes, Pamela R. Chacón-Uscamaita, Patricia J. García

**Affiliations:** 1 Biomedical Sciences Research Institute, School of Medicine, Universidad Ricardo Palma, Lima, Peru; 2 Department of Medicine, Hospital Nacional Hipolito Unanue, Lima, Peru; 3 Tropical Medicine Institute Alexander von Humboldt, Universidad Peruana Cayetano Heredia, Lima, Peru; 4 School of Medicine, Universidad Peruana Cayetano Heredia, Lima, Peru; 5 Instituto Nacional de Enfermedades Neoplasicas, Lima, Peru; 6 Department of Hemotherapy and Blood Transfusion, Instituto Nacional de Salud del Niño San Borja, Lima, Peru; 7 Department of Clinical Pathology and Pathological Anatomy, Hospital Nacional Hipolito Unanue, Lima, Peru; 8 Emerge, Emerging Diseases and Climate Change Research Unit. School of Public Health, Universidad Peruana Cayetano Heredia, Lima, Peru; 9 School of Public Health, Universidad Peruana Cayetano Heredia, Lima, Peru

**Keywords:** COVID-19, Randomized controlled trial, Protocol, Convalescent plasma, Safety, Adverse reactions, Mortality

## Abstract

**Objectives:**

The general objective of this study is to test the hypothesis that administration of convalescent plasma from donors with previous diagnosis of severe COVID-19 pneumonia is safe and associated with a decrease in all-cause in-hospital mortality among hospitalized patients with COVID-19 at 30 days in comparison with standard treatment alone.

The secondary objectives are as follows: (1) to assess the efficacy of convalescent plasma to reduce the length of hospitalization, (2) to assess the efficacy of convalescent plasma to reduce the length of ICU stay, and (3) to assess the efficacy of convalescent plasma on reducing the requirement of invasive mechanical ventilation or ICU stay.

**Trial design:**

PERUCONPLASMA is a IIb phase open label, randomized, superiority clinical trial with 1:1 allocation taking place in real life routine clinical practice at public hospitals in Lima, Peru. Participants will be randomized to receive convalescent plasma along with local standard treatment or local standard treatment alone. After allocation, all participants will be followed for a total of 30 days or until hospital discharge, whichever occurs first.

**Participants:**

The population for the study are patients with severe disease with a confirmed laboratory test for SARS-CoV-2 infection hospitalized in 3 tertiary-care hospitals in Lima, Peru.

Subjects are eligible for the trial if they meet all of the following inclusion criteria:

1. Age 18 or older
2. Hospitalization due to COVID-19 with laboratory confirmation (either with serologic, molecular, or antigen test along with a compatible clinical presentation)
3. Severe or critical COVID-19 disease

Severe illness was defined by 2 or more of the following:

- Respiratory rate of 22 or more
- Hypoxemia with oxygen saturation equal or less than 93%
- Abnormal blood gas analysis (PaO_2_ < 60 mmHg, PaCO_2_ > 50 mmHg, or Pa/FiO_2_ < 300)

Critical disease was defined by either:

- Mechanical ventilation requirement less than 72 h.
- Shock.

4. Capacity to provide informed consent (patient or patient’s direct relative)
5. Availability of convalescent plasma units compatible with ABO blood type of the subject.

Exclusion criteria:

Subjects are not eligible for the trial if they meet any of the following criteria:

1. Contraindication for transfusion (e.g., prior anaphylaxis, congestive heart failure)
2. Hemodynamic instability (PA < 60 mmHg refractory to vasopressors)
3. Uncontrolled concomitant infections\
4. Stupor or coma
5. Platelets < 50,000/μL or disseminated intravascular coagulation
6. Serum creatinine > 3.5 mg/dL or dialysis requirement
7. Total bilirubin > 6 mg/dL or jaundice of unknown etiology
8. Myocardial infarction or acute coronary syndrome
9. Active or recent (< 7 days) intracranial hemorrhage
10. Pregnancy

Donors:

The donors have to meet the following criteria: male between 30 and 60 years with a previous diagnosis of severe COVID-19-associated pneumonia within the last 3 months, with resolution of symptoms of at least 28 days. The rationale for including donors with severe disease is to maximize the probability of collecting convalescent plasma units with high titer of neutralizing antibodies, as the technology to measure this specific type of antibodies is not routinely available in Peru. Aliquots of plasma will be stored for future quantification of neutralizing antibodies.

**Intervention and comparator:**

Convalescent plasma from donors with previous severe COVID-19 is the investigational medical product.

The experimental group will receive 1 to 2 units of 200 to 250 ml of convalescent plasma along with local standard treatment. The control group will receive local standard treatment alone. The participants randomized to plasma will have evaluations at 6 h and 24 h to specifically evaluate possible post transfusion events. All the participants will be evaluated at day 3, day 7, and day 30 after enrolment.

**Main outcomes:**

Safety outcome:

- Incidence of serious adverse reactions related to convalescent plasma transfusion within 24 h after convalescent plasma administration.

Efficacy outcomes:

- Mortality from any cause during hospitalization at 30 days post randomization.
- Length of hospitalization at 30 days post randomization or until hospital discharge.
- Duration of mechanical ventilation at 30 days post randomization or until hospital discharge.
- Length of hospitalization in an intensive care unit at 30 days post randomization or until hospital discharge.

Exploratory:

- Oxygen requirement evolution at days 3 and 7.
- Score Sequential Organ Failure Assessment (SOFA) evolution at days 3 and 7.
- Dynamics of inflammatory marker (lymphocyte, C-reactive protein (CRP), D-dimer, lactate dehydrogenase (LDH)) evolution at days 3 and 7.
- Proportion of patients progressing to multi-organ failure at 30 days post randomization or until hospital discharge.
- Proportion of transfusion related adverse reactions at 30 days post randomization or until hospital discharge.

**Randomization:**

Randomization will be carried out within the electronic case report form (eCRF) in 1:1 ratio (receive plasma/control) in a randomization process established by blocks of size 2, 4, and 6. Allocation to the treatment arm of an individual patient will not be available to the investigators before completion of the whole randomization process. Randomization blocks will be performed with “ralloc”, Stata’s randomization process v.16.0. Randomization through the eCRF will be available 24 h every day.

**Blinding (masking):**

Both the participants and study staff will be aware of the allocated intervention.

Blinded statistical analysis will be performed.

**Numbers to be randomized (sample size):**

The sample size was calculated using the Fleiss formula with continuity correction to detect a mortality reduction from 50 to 20% between the two treatment arms with a confidence level of 95% and a power of 80%. Based on this information, a total of 45 patients per arm would be needed. After adjustment for a drop-out rate of 10% after enrolment, a total of 50 patients per arm (100 patients in total) will be enrolled.

**Trial status:**

Current protocol version: 5.0 dated January 04, 2021.

Recruitment started on September 21, 2020, and is expected to finish by the end of March 2021.

**Trial registration:**

- Peruvian Register of Clinical Trials (REPEC) ID: PER-016-20, registered on June 27, 2020.
- Clinicaltrials.gov ID: NCT04497324, registered on August 4, 2020.

**Full protocol:**

The full protocol is attached as an additional file, accessible from the Trials website (Additional file 1). In the interest in expediting dissemination of this material, the familiar formatting has been eliminated; this letter serves as a summary of the key elements of the full protocol.

**Supplementary Information:**

The online version contains supplementary material available at 10.1186/s13063-021-05189-6.

## Supplementary Information

**Additional file 1.**

## Data Availability

Collected de-identified data of the trial will be shared with other studies on the same topic upon relevant requests.

