# Supplementary material for: Randomized clinical trial to evaluate safety and efficacy of convalescent plasma use among hospitalized patients with COVID-19 (PERUCONPLASMA): a structured summary of a study protocol for a randomized controlled trial
Source: Trials. 2021 May 17;22:342. doi: 10.1186/s13063-021-05189-6 (PMC8127286; doi:10.1186/s13063-021-05189-6)
Supplement: Supplementary file 1 — Additional file 1. [file 13063_2021_5189_MOESM1_ESM.pdf]

Versión
5.0
04 de enero de 2021

PERUCONPLASMA:
ENSAYO CLÍNICO ALEATORIZADO PARA LA EVALUACIÓN DE LA SEGURIDAD Y EFICACIA DEL USO DE PLASMA CONVALECIENTE EN PACIENTES HOSPITALIZADOS CON COVID-19

Protocolo

Investigadores Principales
Patricia García Funegra
Fiorella Krapp López

Co-Investigadores
Alonso Soto Tarazona
Luis Enrique Gerardo Argumanis Sánchez
Martin Montes Delgado
Patricia Liliana García López
Priscilla Karina Altamirano Cáceres
Johan Alexander Azañero Haro
Lucía Esther Salazar Salas

ÍNDICE 1
ABREVIATURAS……………………………………………………………………2
1. PLANTEAMIENTO DEL PROBLEMA 5
1.1. Descripción del problema de investigación 5
1.2. Justificación y relevancia 5
1.3. Limitaciones y viabilidad de la investigación 6
2. ANTECEDENTES 9
3. OBJETIVOS, CRITERIOS DE VALORACIÓN O RESULTADOS E HIPÓTESIS ESPECÍFICAS. 12
3.1. Objetivos: 12
3.2. Criterios de valoración 12
3.3. Hipótesis 12
4. DISEÑO Y PROCEDIMIENTOS CLÍNICOS DEL ESTUDIO 13
5. CRITERIOS DE SELECCIÓN 14
5.3.1. Interrupción de la administración del producto del estudio 15
5.3.2. Interrupción de la participación en el protocolo 15
5.4. Criterios para poner en pausa y reanudar el estudio 15
6. PROCEDIMIENTOS Y EVALUACIONES CLÍNICAS 15
7. SEGURIDAD Y EVENTOS ADVERSOS 19
8. CONSIDERACIONES ESTADISTICAS 20
9. RECOLECCIÓN DE DATOS Y MONITOREO DEL ENSAYO CLÍNICO 21
10. CONSIDERACIONES ÉTICAS 22
10.1. Consentimiento informado 22
10.2. Confidencialidad del sujeto 22
10.3. Riesgos y beneficios 22
10.4. Uso de muestras y datos 23
10.5. Compensación 23
10.6. Medidas de prevención de contagio por COVID-19 23
10.7. Medidas para evitar la saturación del sistema de salud 23
11. PUBLICACIÓN DE LOS RESULTADOS 23
12. REFERENCIAS 24
Anexo 1: Consentimiento Informado para donación de plasma convaleciente COVID-19 27
Anexo 2: Consentimiento Informado para recepción de plasma convaleciente COVID-19 28

ABREVIATURAS:

Abreviatura	Término
ABO	Sistema de grupo sanguíneo que incluye los grupos A, B, O y AB
CBC	hemograma completo (complete blood count)
CDC	Centros para el Control y la Prevención de Enfermedades (Centers for Disease Control and Prevention)
CNTEI	Comité Nacional Transitorio de Ética en Investigación
cGMP	buenas prácticas de fabricación actuales (current good manufacturing practices)
cm	Centímetro
COVID19	Infección por el nuevo coronavirus 2019 (SARS-CoV-2)
EA	evento adverso
EAS	evento adverso serio
ELISA	ensayo por inmunoabsorción ligado a enzimas (enzyme-linked immunosorbent assay)
INS	Instituto Nacional de Salud
GCLP	buenas prácticas clínicas de laboratorio (good clinical laboratory practices)
BPC	Buenas prácticas clínicas (good clinical practices)
H0	hipótesis nula
HLA	antígeno leucocitario humano (human leukocyte antigen)
HTLV-1	virus linfotrópico humano tipo 1
EV	endovenosa; por vía endovenosa
IB	manual del investigador (investigator’s brochure)
IC	intervalo de confianza
ICF	formulario de consentimiento informado (informed consent form)
ICH	Consejo Internacional sobre Armonización de los Requisitos Técnicos para el Registro de Productos Farmacéuticos para Uso Humano (International Council for Harmonisation)
IgM	inmunoglobulina M
IgG	inmunoglobulina G
IP	investigador principal
ITT	intención de tratar (intent-to-treat)
MedDRA®	Diccionario Médico para las Actividades Reguladoras (Medical Dictionary for Regulatory Activities)
MERS	Síndrome respiratorio de Oriente Medio
mITT	intención de tratar modificada (modified intent-to-treat)
NAb	anticuerpo neutralizante (neutralizing antibody)
NIH	Institutos Nacionales de Salud (National Institutes of Health)
OMS	Organización Mundial de la Salud
OPS	Organización Panamericana de la Salud

PCR	reacción en cadena de la polimerasa (polymerase chain reaction)
PP	por protocolo
RT-PCR	reacción en cadena de la polimerasa-transcriptasa inversa (reverse transcription-polymerase chain reaction)
SAP	plan de análisis estadístico (statistical analysis plan)
SARS	síndrome respiratorio agudo grave (severe acute respiratory syndrome)
SUSAR	presuntas reacciones adversas serias e inesperadas (serious and unexpected suspected adverse reactions)
TRALI	injuria pulmonar aguda relacionada a transfusión
TACO	sobrecarga circulatoria asociada a transfusión
UCI	Unidad de cuidados intensivos
UP	problema inesperado (unanticipated problem)
VIH	Virus de inmunodeficiencia humana

RESUMEN

**Título del estudio:**	**PERUCONPLASMA: Ensayo clínico aleatorizado para la evaluación de la seguridad y eficacia del uso de plasma convaleciente en pacientes hospitalizados con COVID-19**
**Problema a Investigar:**	El uso de hemocomponentes y hemoderivados ha sido reportado como una medida de salvataje en pacientes con otras enfermedades virales, como Ébola, MERS e Influenza H1N1. Recientes estudios observacionales, incluyendo uno de 5000 pacientes, sugieren que el plasma de pacientes recuperados de COVID-19 puede utilizarse de manera segura y podría tener un efecto positivo en la reducción de la mortalidad en pacientes con enfermedad severa o crítica
**Justificación y Relevancia:**	Dada la elevada mortalidad asociada a enfermedad severa por COVID-19, y el creciente número de pacientes que la presentan, urge encontrar una terapia efectiva y que pueda ser utilizada con seguridad en el Perú.
**Objetivo:**	**Objetivo principal** es evaluar la seguridad de la administración de plasma convaleciente en pacientes hospitalizados por COVID-19, reportando la proporción de pacientes que desarrollan eventos adversos graves relacionados al tratamiento del estudio. **El objetivo secundario** es evaluar la eficacia de la administración de plasma convaleciente en la reducción de la mortalidad de pacientes hospitalizados por SARS-CoV-2 en comparación con el estándar de atención local. **Los objetivos exploratorios** del estudio son evaluar el efecto del plasma convaleciente en la evolución clínica del paciente, duración de ventilación mecánica, tiempo de estadía en UCI, evolución de los parámetros ventilatorios y parámetros inflamatorios.
**Metodología:**	Este estudio es un ensayo clínico de fase 2b, abierto, aleatorizado 1:1, para evaluar la seguridad y la eficacia del uso de plasma convaleciente en pacientes con infección por SARS-CoV-2. De dos brazos el grupo experimental recibirá plasma convaleciente más el estándar de atención local y el grupo control recibirá el estándar de atención local.<br><br>Consta de 2 fases:<br><br><u>Fase de implementación</u>: Se implementará el sistema de promoción y captación de donantes, colecta, procesamiento y almacenamiento de plasma convaleciente y alícuotas para estudios futuros. Se espera colectar plasma de al menos 10 pacientes recuperados de COVID-19 de manera centralizada, para iniciar la fase de uso de plasma.<br><br><u>Fase de uso de plasma convaleciente:</u> El grupo experimental recibirá 1 a 2 unidades de 200ml a 250ml de plasma convaleciente (ABO compatible), obtenido según criterios establecidos, para el manejo de pacientes hospitalizados por COVID-19. Se monitorizará la seguridad del uso de este plasma y algunos indicadores de eficacia clínica. Se incluirá a pacientes mayores de 18 años hospitalizados, con diagnostico confirmado por una prueba de laboratorio positiva para COVID-19, con enfermedad severa o crítica, y que cuenten con consentimiento informado proporcionado por paciente o familiar directo.

1. PLANTEAMIENTO DEL PROBLEMA

1.1. Descripción del problema de investigación

El 11 de marzo de 2020, la OMS declara oficialmente la pandemia por SARS-CoV-2. Hasta el momento, se han reportado más de 2 millones de casos a nivel mundial y más de 135 000 muertes a causa de este virus (1).

Actualmente no existen tratamientos probados para la neumonía asociada a la enfermedad por coronavirus (COVID-19) cuyo agente etiológico es el SARS-CoV-2. Aproximadamente 15% de los pacientes diagnosticados con COVID-19 presenta enfermedad severa, requiriendo hospitalización, y 5% presenta enfermedad crítica, definida como falla respiratoria que requiere ventilación mecánica, shock o falla multiorgánica, requiriendo manejo en una unidad de cuidados intensivos (UCI). La mortalidad en este último grupo de pacientes ha sido reportada cercana al 50% (2).

El 5 de marzo, se reportó el primer caso de COVID-19 en Perú. Al 13 de Mayo, el número de casos asciende a más de 76 000, con 6 979 pacientes requiriendo hospitalización y 806 requiriendo atención en la unidad de cuidados intensivos (UCI) (3).

1.2. Justificación y relevancia

Dada la elevada mortalidad asociada a enfermedad severa por COVID-19, y el creciente número de pacientes que la presentan, urge encontrar una terapia efectiva y que pueda ser utilizada con facilidad en el Perú.

Estudios previos han demostrado en otras enfermedades respiratorias virales, incluidas aquellas causadas por otros coronavirus han reportado que la transfusión de plasma convaleciente es eficaz en reducir la mortalidad en pacientes con enfermedad severa (4-10). En base a estos hallazgos, la OMS publicó en el 2017 un documento de posición favorable al uso de plasma convaleciente como elemento de respuesta a brotes de virus emergentes, recomendando que su uso clínico se maneje como terapia experimental o bajo un protocolo MEURI (por sus siglas en inglés, *monitored emergency use of unregistered and experimental interventions)* y manteniendo estrictos estándares para prevenir la enfermedades transmitidas por transfusión (11, 12).

Estudios preliminares no controlados en pacientes con enfermedad severa y crítica por COVID-19, han reportado que la transfusión de plasma convaleciente mejora la evolución clínica y no se asocia a efectos adversos severos. En base a estos hallazgos, el uso de plasma de pacientes recuperados ha sido aceptado como una posible alternativa para tratar casos graves por Covid-19 por la FDA de EE.UU. (13) y la OMS (14), dentro del marco de ensayos clínicos o de un protocolo MEURI. De tal manera, existen ya varios protocolos de investigación y protocolos de uso expandido de plasma convaleciente en Europa y EE.UU. (15-17), algunos de los cuales se resumen en la **Tabla 1**. Más aún, un reporte reciente de la evaluación de seguridad realizado en los primeros 5 000 pacientes enrolados en uno de estos protocolos muestra que la administración de plasma convaleciente en pacientes con enfermedad severa es segura, con una incidencia de <1% de eventos adversos serios. En este reciente reporte también se observa una mortalidad a 7 días de 14.9% para el total de sujetos y de 16.7% para aquellos que se encontraban en UCI (18). Estas tasas de mortalidad se muestran relativamente bajas en comparación con las tasas de mortalidad de 15-50% para pacientes hospitalizados y >50% para pacientes en UCI reportadas en distintos estudios previos (19, 20).

Por estos motivos, es imperativo que se estudie la seguridad y eficacia del uso de plasma convaleciente en el manejo de pacientes hospitalizados por COVID-19 en el Perú, siendo la mejor manera de evaluar ello a través de un ensayo clínico aleatorizado, las normativas actual promueve el desarrollo de ensayos clínicos de importancia en salud pública, en escenarios de emergencias como los actuales, y mejor aun cuando se cuenta con la

evidencia acumulada internacionalmente al momento sobre la seguridad y potencial beneficio del plasma convaleciente, consideramos que es de vital importancia que se realice este estudio nacional.

Por este motivo, planteamos realizar este estudio de fase 2, a efectos de probar la seguridad y eficacia de intervenciones aún en fases de experimentación en un menor número de sujetos, con el fin de demostrar su factibilidad y que ésta sea registrada como una terapia a considerar en el manejo de los pacientes hospitalizados por COVID-19 (12).

1.3. Limitaciones y viabilidad de la investigación

La cuantificación de anticuerpos neutralizantes requiere del cultivo del virus SARS-CoV-2, para lo cual se requiere de un laboratorio de nivel BSL-3 y personal altamente capacitado en este procedimiento. Dada la coyuntura actual de emergencia nacional a causa de la pandemia, la factibilidad de realizar este tipo de procedimiento es muy baja y causaría una demora significativa en la implementación del estudio y evaluación de los potenciales beneficios de esta alternativa de tratamiento. Por este motivo, se plantean criterios estrictos y basados en la evidencia actual y en experiencias internacionales, para seleccionar a los potenciales donantes con mayor probabilidad de tener plasma convaleciente con niveles altos de este tipo de anticuerpos. Así mismo se plantea almacenar, con el consentimiento del donante, alícuotas del plasma colectado para en un futuro se pueda realizar estas mediciones. A medida que aparezcan opciones para la cuantificación de los anticuerpos neutralizantes esta estrategia podrá ser incorporada. Lo que se está haciendo en otros países y haremos en este estudio es guardar una alícuota para posteriores estudios de anticuerpos neutralizantes.

En el Perú, el uso de plasma convaleciente humano podría ser una opción para el tratamiento de COVID-19, ya que se cuenta con un número elevado de potenciales donantes, considerando que a la fecha más de 2000 personas con enfermedad moderada o severa por COVID-19 han sido dadas de alta luego de haber estado hospitalizadas, y más de 6000 se encuentran hospitalizadas (3).

Tabla 1. Comparación de los protocolos disponibles en distintos países

**Institución**	**UCLA**	**Mayo Clinic**	**Johns Hopkins**	**Ministerio de Sanidad de España**
**Título**	UCLA Convalescent Plasma Initiative: Compassionate Use Treatment Protocol	Expanded Access to Convalescent Plasma for the Treatment of Patients with COVID-19	Convalescent Plasma to Treat Coronavirus - Associated Severe Pulmonary Complications: A Feasibility Study Assessing the Safety of Multiple Doses of Anti-SARS-CoV-2 Plasma in Mechanically Ventilated Intubated Patients with Respiratory Failure due to COVID-19	Recomendaciones para la obtención de plasma de donantes convalecientes de COVID-19
**Código**	CCPP-19	20-003312	CPPulm-001	
**Diseño**	Estudio piloto de factibilidad de uso compasivo	Expanded access program	Estudio de brazo único	No es estudio de investigación
**Objetivo primario**	Evaluar seguridad y factibilidad	Proporcionar acceso a plasma convaleciente COVID-19	Evaluar factibilidad	NA
**Objetivos secundarios**	Evaluar eficacia en reducir severidad de infección por SARS-CoV-2, basado en escala ordinal de 7 puntos y en la incidencia de paro cardio-respiratorio, transferencia a UCI, tipo y duración de soporte ventilatorio, mortalidad y tiempo en UCI, mortalidad intrahospitalaria y tiempo de hospitalización, días libres de ventilados, mortalidad a 28 días	Evaluación de la seguridad (incidencia de efectos adversos serios)<br>Exploratorios: tiempo de hospitalización, N días en UCI, N días en vent mecanica, sobrevida al alta	Evaluar sobrevida	NA
**Población**	- Infección confirmada por preba de laboratorio<br>-Enfermedad severa o crítica, con o sin ventilación mecánica	- Infección confirmada por RT-PCR<br>-Enfermedad severa o critica, con o sin ventilación mecánica; o medico tratante considera que está	- Infección confirmada por RT-PCR<br>- con ventilación mecánica	NA

		en riesgo de evolucionar a esa severidad		
**Donantes**	- Infección confirmada por RT-PCR<br>- Asintomático por > 14 días<br>- Negativización de RT-PCR<br>- ≥18 años	Según FDA:<br>- Infección confirmada por RT-PCR o serología<br>- Resolución de síntomas por ≥ 28 días o asintomático por ≥14 días y RT-PCR negativo<br>- Hombre o mujer que nunca ha gestado<br>- ≥18 años	- Varones recuperados con ≥14 días asintomáticos (no indican nada mas)	- Infección confirmada por prueba de laboratorio (no especifica cual)<br>- Asintomático por ≥14 días<br>- 2 RT-PCR negativos<br>- ≥18 años
**Intervención**	1-2 unidades	1 unidad	1-2 unidades en los días 0, 2, 4, 6, y 8	NA
**Seguimiento**	90 días	Hasta alta hospitalaria	60 días	NA

2. ANTECEDENTES

El uso de hemocomponentes y hemoderivados ha sido reportado como una medida de salvataje en pacientes con otras enfermedades virales. Un estudio no randomizado en pacientes con Ebola mostró un efecto benéfico en la sobrevida con la administración de sangre total convaleciente (4)**.** El plasma convaleciente también se ha utilizado satisfactoriamente en el manejo de presentaciones severas de otras enfermedades respiratorias virales, tales como influenza A (H1N1), SARS y MERS (ambos pertenecientes a la familia de coronavirus), con resultados positivos en cuanto a eficacia y seguridad ***(5-10)***. Un meta-análisis de 32 estudios con pacientes con infección severa por SARS-CoV-1 o por influenza, mostró una reducción significativa del odds combinado para mortalidad en el grupo de pacientes que recibieron plasma convaleciente, comparado con placebo o con manejo no farmacológico (odds ratio, 0.25; IC al 95%, 0.14–0.45) (9)**.**

La experiencia con epidemias previas por coronavirus SARS-CoV-1 muestra que el suero convaleciente contiene anticuerpos neutralizantes los cuales pueden ser cuantificados (10)**.** Así mismo, un estudio previo con MERS-CoV reportó que el uso de plasma convaleciente solo fue efectivo en los casos en los que el plasma convaleciente contenía títulos de anticuerpos neutralizantes mayores a 1:80 (8). También se ha descrito que en pacientes con infección por SARS-CoV-1, los anticuerpos neutralizantes comienzan a disminuir gradualmente a partir de los 4 meses post-enfermedad, encontrándose solo en 16% de los pacientes a los 36 meses post-enfermedad (21). En pacientes con infección por SARS-CoV-2, se ha descrito que la mediana de tiempo desde el inicio de síntomas hasta la detección de IgG es 14 días [RIQ: 10-18] (22), sin embargo, se desconoce cómo varían específicamente los niveles de anticuerpos neutralizantes en estos pacientes. Un estudio reciente en el que se tamizó a 40 pacientes recuperados de SARS-CoV-2 con al menos 3 semanas post-enfermedad, se encontró que 39 presentaron títulos de anticuerpos neutralizantes mayores a 1:160 (23).
Aún no se conoce qué grupo de pacientes tiene mayor probabilidad de producir altos niveles de anticuerpos neutralizantes. Un reciente estudio en 175 pacientes recuperados y hospitalizados con síntomas leves a moderados, reportó una alta variabilidad en los niveles de producción de anticuerpos neutralizantes (24). En este estudio se encontró que 30% de los pacientes no presentaron anticuerpos neutralizantes, incluso después de 4 semanas del inicio de síntomas. Entre los pacientes que sí tuvieron anticuerpos neutralizantes detectables, se encontró también una alta variabilidad. Interesantemente, pacientes adultos (40-59 años) y adultos mayores (60-85 años) tuvieron títulos más altos que los adultos jóvenes. Además reportaron una correlación entre el nivel de anticuerpos neutralizantes y el nivel de PCR (correlación positiva) y el conteo linfocitario de sangre periférica (correlación negativa). Elevados niveles de IgG anti-RBD y IgG anti-S2 también correlacionaron con niveles elevados de anticuerpos neutralizantes (24).

A la fecha, se han publicado 7 estudios no controlados, del tipo serie de casos, reportando un total de 5033 pacientes con COVID-19 severos tratados con plasma convaleciente (18, 23, 25-29). Estos estudios sugieren que el uso de plasma convaleciente es seguro, ya que solo en el estudio de 5000 pacientes se reportó la ocurrencia de eventos adversos serios, y estos se presentaron en <1% de los pacientes En estos estudios también se reportó en una proporción importante de pacientes una mejora clínica y laboratorial post-transfusión, tales como mejora en parámetros ventilatorios, marcadores de inflamación, y reducción de la carga viral post-transfusión. Estos estudios también sugieren una posible reducción de la mortalidad, con 5 de las 6 series de casos pequeñas reportando 0% de mortalidad, y con el estudio de 5000 pacientes reportando una mortalidad de 16.4% en los pacientes de UCI. Esta mortalidad es significativamente menor a la mortalidad en cohortes históricas de alguno de los mismos hospitales participantes 30% - 50% para pacientes en UCI. La **Tabla 2** resume los métodos y hallazgos más importantes de estos estudios.

En cuanto a la seguridad del uso de plasma convaleciente en pacientes con COVID-19, existen riesgos teóricos asociados a su uso, tales como la exacerbación mediada por anticuerpos descrita en otras infecciones virales, incluyendo otros coronavirus (30). El uso de altos niveles de anticuerpos neutralizantes específicos para SARS-CoV-2 en teoría minimiza este riesgo. Esto es apoyado por los resultados mencionados anteriormente de los 5 estudios publicados sobre uso de plasma convaleciente en pacientes con COVID-19 (18, 23, 25-29).

Otro riesgo teórico es la atenuación de la respuesta inmunitaria, lo cual podría resultar en vulnerabilidad a una segunda infección por SARS-CoV-2. A pesar que este riesgo no ha sido aún evaluado, se postula que el beneficio de reducción la alta morbi-mortalidad asociada a enfermedad crítica por COVID-19 supera este potencial riesgo. No obstante, es algo que debe ser evaluado.

Finalmente, existen otros riesgos relacionados a la transfusión de hemocomponentes y hemoderivados, tales como plasma. Estos riesgos incluyen la anafilaxis y reacciones alérgicas, injuria pulmonar aguda relacionada a transfusión (TRALI, por sus siglas en inglés), sobrecarga circulatoria asociada a transfusión (TACO, por sus siglas en inglés), transmisión de infección tales como VIH, HTLV-1, HBV, HCV, entre otros. Algunas de estas complicaciones pueden ser evitadas con medidas estándares de tamizaje de donantes. Adicionalmente, se conoce que el TRALI está causado anticuerpos anti-HLA y anti-neutrófilos del donante, y que los donantes de sexo masculino tienen menor chance de tener estos anticuerpos.

Tabla 2. Resumen de los 7 estudios publicados a la fecha sobre el uso de plasma convaleciente en pacientes con COVID-19

Autor	País	Intervención			Población de estudio				Variables			Resultados	
		Criterios para donantes	Características de plasma	Dosis	N° de pacientes	Criterios de selección	Tiempo de administración	Tratamiento concomitante	Clínicas	Laboratoriales	Tiempo de seguimiento post-transfusión	Efectos adversos	Condición al termino del estudio
Shen et al.[15]	China	18-60 años<br>Confirmación SARS-CoV-2 por RT-PCR<br>Asintomáticos por ≥10 días<br>Un RT-PCR negativo<br>ELISA con título de Ab ≥ 1:1000<br>Ab neutralizante >1:40	Títulos de IgG e IgM a RBD (ELISA) 1:1800-1:16200<br>Ab neutralizantes 1:80 – 1:480	2 dosis de 200ml-250ml transfundidas el mismo día	5	Edad 30-70 años<br>4/5 sin comorbilidades<br>Críticos en ventilación mecánica<br>RT-PCR positivo	14 a 24 días post-inicio de síntomas	6/10 recibió metilprednisolona	Temp<br>SOFA<br>PaO2/FiO2<br>Cambios en TAC<br>Días de vent mecánica	Proxy carga viral (Ct de RT-PCR de hisopado NF)<br>PCR<br>IL-6<br>Pro-calcitonina	12 días	Ninguno	0 fallecidos<br>3 de alta<br>2 en ventilación mecánica
Duan et al.[13]	China	Afebril por ≥3 días<br>Resolución de síntomas respiratorios<br>Dos RT-PCR negativos separados por 1 día<br>3 semanas post inicio de síntomas<br>4 días post alta de hospital<br>Ab neutralizante 1:160	Ab neutralizantes >1:640<br>Tratamiento con luz UV y azul de metileno	1 dosis de 200ml	10	Edad 34-78 años<br>4/10 con comorbilidades<br>Neumonía severa (Sat O2≤93%), 3/10 en ventilación mecánica<br>RT-PCR positivo	11 a 20 días post-inicio de síntomas	Antivirales (Lopinavir/ritonavir, favipiravir, darunavir, IFN-alfa)<br>Metilprednisolona	Temp<br>SatO2<br>Cambios en TAC	Linfocitos<br>PCR<br>TGO/TGP<br>Bil Totales	4 días	1 paciente con rash facial evanescente	0 fallecidos<br>3 de alta<br>7 en ventilación mecánica
Ye et al.	China	Afebril por ≥3 días<br>Resolución de síntomas respiratorios<br>Dos RT-PCR negativos separados por 1 día<br>3 semanas post inicio de síntomas<br>Seropositivo para SARS-CoV-2	RT-PCR negativo para SARS-CoV-2	1 a 3 dosis de 200ml dadas cada 2-3 días	6	Persistencia de positividad de RT-PCR<br>Neumonía (ninguno estaba en UCI o en VM)	>4 semanas post-inicio de síntomas	No descrito	Descripción de mejora clínica y cambios en TAC	RT-PCR	Hasta el alta	Ninguno	0 fallecidos<br>6 de alta
Zhang et al.	China	No se describe	No se describe	1 a 8 dosis de 200ml a 400ml	4	Pacientes críticos con ARDS en VM no invasiva, intubados o en ECMO	11 a 18 días post-admisión	Antivirales (Lopinavir/ritonavir, arbidol, IFN-alfa 2b, ribavirina)<br>Metilprednisolona (1)	Descripción de mejora clínica y cambios en TAC:	Proxy carga viral (Ct de RT-PCR de hisopado NF)	Hasta el alta	Ninguno	0 fallecidos<br>3 de alta<br>1 transferido a C. intermedios
Ahn et al.	Corea	Neumonia bilateral por COVID-19<br>ELISA IgG positivo<br>RT-PCR neg	Elisa IgG OD 0.53-0.58	2 dosis	2	Edad 67-71 años<br>ARDS en VM	7 a 22 días post-inicio de síntomas	Hidroxicloroquina<br>Lopinavir/ritonavir<br>Metilprednisolona	PaO2/FiO2<br>Cambios en TAC<br>Días de vent mecánica	Proxy carga viral (Ct de RT-PCR de hisopado NF)<br>Linfocitos<br>PCR<br>IL-6	20 días	Ninguno	0 fallecidos<br>1 de alta<br>1 extubado
Zeng et al	China	Adultos jovenes<br>1-2 semanas de recuperados<br>RT-PCR neg<br>IgM e IgG positivo	No se describe	1-2 dosis de 200ml	6	31-80 años<br>En UCI	Media de 21 días desde primer RT-PCR positivo	Antivirales, antibióticos, corticoides	Clearance de virus y mortalidad	Duración de positividad para RT-PCR	Hasta el alta	Ninguno	5 fallecidos<br>1 de alta
Richardson et al.	EE.UU.	Según FDA:<br>- InfeccionInfección confirmada por RT-PCR o serología<br>- Resolución de sintomassíntomas por ≥ 28 días o asintomático por ≥14 días y RT-PCR negativo<br>- Hombre o mujer que nunca ha gestado<br>- ≥18 años	No se describe	1-2 dosis de 200ml - 250ml	5000	>18 años<br>Con enfermedad severa o crítica	No se describe	No se describe	Eventos adversos serios (muerte a los 7 días, TACO, TRALI)	N/A	7 días	SAE <1% (n=36), incluyendo mortalidad a las 4 horas 0.3%. 25 fueron consideradas posiblemente asociadas a la transfusión (TACO 7, TRALI 11, Rx severa alérgica 3)	Mortalidad a los 7 días de 14.9%

3. OBJETIVOS, CRITERIOS DE VALORACIÓN O RESULTADOS E HIPÓTESIS ESPECÍFICAS.

3.1. Objetivos:

Objetivo general: Evaluar la seguridad y eficacia del uso de plasma en pacientes hospitalizados por COVID-19 más el estándar de atención local, comparado con el estándar de atención local.

Objetivo Primario: Evaluar la seguridad de la administración de plasma convaleciente en pacientes hospitalizados por COVID-19.

Objetivos Secundarios:. Evaluar la eficacia de la administración de plasma convaleciente en:

- La reducción de la mortalidad de pacientes hospitalizados.
- Duración de la estancia hospitalaria
- La recepción de la ventilación asistida o cuidados intensivos.
- Tiempo de estadía en UCI, y

Objetivos exploratorios: Evaluar los parámetros ventilatorios y parámetros inflamatorios.

3.2. Criterios de valoración

Criterio de valoración primario (seguridad): Incidencia de efectos adversos serios relacionados a la transfusión de plasma convaleciente.

Criterio de valoración secundario (eficacia):

1. Mortalidad por todas las causas
2. Tiempo de estancia hospitalaria
3. Tiempo hasta el momento en que recibió ventilación asistida o cuidado intensivos por primera vez.
4. Duración de ventilación mecánica (días)
5. Tiempo de estadía en UCI

Criterios de valoración exploratorios:

6. Evolución del requerimiento de oxígeno (e.g. FiO2)
7. Evolución de los parámetros de severidad (SOFA)
8. Evolución de los marcadores de inflamación y predictores de mortalidad (linfopenia, PCR, Dímero D, DHL, TGO/TGP, GGT, bilirrubinas totales, albumina,)
9. Proporción de pacientes que desarrolla falla sistémica multiorgánica (diálisis, vasopresores)
10. Proporción de pacientes que desarrolla reacciones asociadas a transfusión basado en definiciones de consenso.

3.3. Hipótesis

El tratamiento con 1-2 dosis de plasma convaleciente con anticuerpos IgG anti-SARS-CoV-2 es seguro y se asocia a una disminución de la mortalidad en pacientes hospitalizados por COVID-19 en comparación con el cuidado estándar.

4. DISEÑO Y PROCEDIMIENTOS CLÍNICOS DEL ESTUDIO

a. Diseño del estudio

Ensayo clínico de fase 2b, abierto, randomizado de dos brazos, con aleatorización 1:1, para la evaluación de la seguridad y eficacia del uso de plasma convaleciente en el tratamiento de pacientes hospitalizados debido a COVID-19, en comparación al uso del estándar de atención local. Consta de 2 fases:

Fase de implementación: Se implementará el sistema de promoción y captación de donantes, colecta, procesamiento y almacenamiento de plasma convaleciente y alícuotas para estudios futuros. Se espera colectar plasma de al menos 10 pacientes recuperados de COVID-19 de manera centralizada, para posteriormente iniciar la fase de uso de plasma.

Fase de uso de plasma convaleciente: El grupo experimental recibirá 1 a 2 unidades de 200ml a 250ml de plasma convaleciente (ABO compatible), obtenido según criterios establecidos, para el manejo de pacientes hospitalizados por COVID-19. Se monitorizará la seguridad del uso de este plasma y algunos indicadores de eficacia clínica. Se incluirá a pacientes mayores de 18 años hospitalizados, con diagnostico confirmado por una prueba de laboratorio positiva para COVID-19, y que cuente con consentimiento informado proporcionado por paciente o familiar directo.

b. Duración esperada

El estudio se llevará a cabo entre los meses de Julio a Diciembre del 2020. La captación de los donantes se realizará en la primera fase, durante los primeros 2 meses del estudio. La segunda fase se iniciará una vez se haya logrado colectar el plasma de al menos 10 donantes. En ésta segunda fase se realizará el enrolamiento de los sujetos de investigación (receptores del plasma) y el seguimiento de éstos por un total hasta el alta hospitalaria o hasta por un total de 30 días.

c. Intervención del estudio

El plasma convaleciente deberá transfundirse a los pacientes seleccionados utilizando procedimientos clínicos estándar de transfusión y únicamente después de obtener el consentimiento informado de su familiar directo.

- La dosis será 1 a 2 unidades de plasma de 200-250 ml. El médico tratante podrá decidir dar una sola unidad de plasma si considera de que existe riesgo de sobrecarga de volumen para el paciente en base a su peso y condición hemodinámica.
- La velocidad de infusión de la unidad se realizará de acuerdo a las condiciones clínicas del paciente, y no deberá ser mayor a 200 ml/hora
- El uso de medicinas pre-transfusión tales como paracetamol o difenhidramina se deja a criterio del médico tratante
- Se deberá monitorizar al paciente durante la totalidad de la transfusión.
- Ante reacciones leves (picazón, quemazón en sitio de infusión) se deberá disminuir la velocidad de infusión o pausar la infusión. Si no se evidencia progresión de los síntomas se podrá continuar con la infusión a menor velocidad, según decisión del médico tratante.

Ante reacciones alérgicas severas, se deberá descontinuar la infusión de inmediato y no ser reiniciada. Algunas de estas reacciones incluyen:

- Manifestaciones mucosa o cutáneas como prurito intenso, eritema generalizado, edema de labios, lengua o úvula
- Compromiso respiratorio: sibilantes, hipoxemia
- Disminución de la presión arterial sistólica a < 90 mmHg o disminución >30% de la presión basal; o caída la presión diastólica en >30% de la presión basal
- Taquicardia que incrementa a >130 latidos por minute; o bradicardia con <50 latidos

por minuto.
- Cualquier otro signo que en base al juicio del médico tratante requiere descontinuación de la transfusión**.**

Toda reacción observada deberá ser registrada en la historia clínica del paciente y en el CRF electrónico (implementado en REDCap)

5. CRITERIOS DE SELECCIÓN

5.1. Criterios para selección de donantes:

Los donantes de plasma convaleciente serán aquellos que cumplan con los siguientes criterios:

- Cumplir con los requisitos de donación de sangre de acuerdo a normas vigentes (R.M. Nº339-2020-MINSA,R.M N° 241-2018-MINSA, y las que las reemplacen)
- Haber estado hospitalizado por enfermedad moderada o severa causada por COVID-19 o requerimiento de oxígeno domiciliario en los últimos 3 meses y que ésta haya sido confirmada por RT-PCR
- Ser asintomático por lo menos 28 días.
- Tener entre 30 y 60 años
- Ser de sexo masculino
- Cumplir con las pruebas de tamizaje serológico para donantes de acuerdo a norma vigente
- Detección de IgG anti-SARS-CoV-2 al momento del tamizaje
- De poder realizarse, título de anticuerpos neutralizantes anti-SARS-CoV-2 mayor o igual a 1:160
- Firma del Consentimiento Informado del estudio para donación (**Anexo 1**)
- Firma de la Declaración Jurada (R.M. Nº339-2020-MINSA) y Consentimiento Informado (R.M. Nº614-2004/MINSA) de PRONAHEBAS

5.2. Criterios para selección de receptores:

a) Criterios de inclusión:

- Edad igual o mayor a 18 años
- Paciente hospitalizado con pruebas de laboratorio positiva para COVID-19 (molecular, serológica o de antígeno, acompañado de cuadro clínico típico a criterio de médico tratante)
- Enfermedad severa o crítica por COVID-19

  Enfermedad severa definida como 2 o más de los siguientes criterios:
  - Distress respiratorio con FR >22
  - Hipoxia con Sat O2 ≤93%
  - Gasometría arterial anormal (PaO2 <60mmHg, PaCO2 >50mmHg o PaO2/FiO2 <300

  ó

  Enfermedad crítica definida como presencia de cualquiera de las siguientes:
  - Insuficiencia respiratoria con requerimiento de ventilación mecánica menor a 72 horas
  - Shock
- Paciente o familiar directo capaz de brindar consentimiento informado (**Anexo 2**)
- Disponibilidad de unidades de plasma convaleciente compatible con el grupo sanguíneo ABO del participante

b) Criterios de exclusión:

- Contraindicación para la transfusión (sobrecarga severa de volumen, historia de anafilaxia a hemocomponentes)
- Inestabilidad hemodinámica definida como PA<60 <u>refractaria</u> a uso de

vasopresores

- Infecciones concomitantes no controladas
- Paciente con estupor o coma
- Plaquetas <50,000/µL o coagulación intravascular diseminada Creatinina sérica >3.5 mg/dL o requerimiento de diálisis
- Bilirrubina total >6 mg/dL o ictericia no explicable
- Síndrome coronario agudo o injuria miocárdica aguda.
- Sangrado intracraneal activo o en los últimos 7 días
- Gestación

5.3. Criterios para la interrupción de las administraciones del producto o de la participación del sujeto

Las decisiones de interrumpir la administración del producto o de interrumpir la participación de un sujeto en el protocolo estarán a cargo del IP del centro o su delegado (sub-investigador).

5.3.1. Interrupción de la administración del producto del estudio

Se puede interrumpir la administración del producto del estudio de un sujeto por los siguientes motivos:

- El sujeto presenta una reacción alérgica severa, incluyendo: Manifestaciones mucosas o cutáneas como eritema generalizado, edema de labios, lengua o úvula; compromiso respiratorio: sibilantes, hipoxemia; disminución de la presión arterial sistólica a < 90 mmHg o disminución >30% de la presión basal; o caída la presión diastólica en >30% de la presión basal; taquicardia que incrementa a >130 latidos por minute; o bradicardia con <50 latidos por minuto; o
- Cualquier otro signo que en base al juicio del médico tratante requiere descontinuación de la transfusión.

5.3.2. Interrupción de la participación en el protocolo

La participación del sujeto en el protocolo se puede interrumpir por los siguientes motivos:

- El sujeto fallece
- El familiar que firmó el consentimiento informado o el paciente solicita su salida del estudio.
- El patrocinador del EC o las autoridades reguladoras interrumpen el protocolo

5.4. Criterios para poner en pausa y reanudar el estudio

El enrolamiento y administración del tratamiento de estudio será detenido y una revisión ad hoc será iniciada en los siguientes casos:

- Ocurrencia de muerte de un sujeto dentro de la primera hora de infusión de plasma.
- Ocurrencia de una reacción alérgica/anafiláctica potencialmente mortal, manifestada por broncoespasmo con o sin urticaria o angioedema, con requerimiento de soporte hemodinámico con vasopresores o ventilación mecánica.
- Ocurrencia de un patrón de eventos clínicos o de laboratorio que el monitor de seguridad independiente o el comité de monitoreo de seguridad consideran que podría estar asociado con el producto del estudio y que puede representar una seria amenaza a la seguridad de los sujetos participantes.
- Cualquier otro evento que bajo el juicio del investigador, pueda representar un serio riesgo para la seguridad de los sujetos participantes.

6. PROCEDIMIENTOS Y EVALUACIONES CLÍNICAS

Todos los procedimientos correspondientes a la selección, extracción, procesamiento, almacenamiento, distribución y transporte de plasma de donantes convalecientes de

COVID-19 deberán alinearse con las Disposiciones Generales y Específicas de la Directiva Sanitaria que establece los lineamientos técnicos para la obtención de plasma de donantes convalecientes de la enfermedad del coronavirus 2019 (COVID-19), aprobada con R.M. Nº339-2020-MINSA, y a los documentos técnicos que la actualicen. Así mismo, cada uno de estos procedimientos cuenta con un POE, los cuales se anexan a este protocolo.

6.1. Fase de implementación (captación, colecta, procesamiento y almacenamiento de plasma)

Esta fase se centralizará en las instalaciones del Instituto Nacional de Salud del Niño de San Borja. Se contará con una central telefónica, un área para la recepción de los posibles donantes, otra para la colecta de plasma, y otra para el procesamiento y almacenamiento del plasma convaleciente. El proceso para toda esta fase esta dividida en los siguientes pasos:

a) Promoción y captación de posibles donantes

Promoción y captación en la comunidad

- Se dispondrá de una central telefónica para recibir llamadas de consulta de potenciales donantes y un equipo de personas entrenadas en contestar estas llamadas.

- Se realizará una invitación a todos aquellos pacientes recuperados de COVID-19 para que llamen a esta central telefónica si están interesados en ayudar con la donación de su plasma. Esta invitación se difundirá a través de diversos medios, incluidos radio, televisión, prensa escrita, y redes sociales.

Promoción y captación intrahospitalaria

- Los médicos tratantes de pacientes con COVID-19 que estén siendo dados de alta, les informarán sobre la posibilidad de ayudar a través de la donación de su plasma. De estar interesados, se tomarán los datos del paciente y éstos se enviarán de manera encriptada al equipo de captación de donantes, para que contacten a los pacientes en los siguientes días.

- Se pondrán afiches en los hospitales y clínicas solicitando donantes y especificando los requisitos para ser un donante y dirigiendo a los interesados a llamar a la central telefónica designada para la captación de posibles donantes

b) Selección de posibles donantes

Tamizaje telefónico

- Cuando un potencial donante contacte a la central telefónica y manifieste su deseo de donar su plasma, se procederá a realizar un tamizaje telefónico utilizando el Check-list – Candidato a donante a través de REDCap, en el cual se verificará si cumple con los criterios de inclusión y exclusión listados en la sección 5.1.

- La entrevista telefónica, así como el llenado del check-list será realizado por personal entrenado en estos procedimientos.

Tamizaje presencial

Si el potencial donante cumple con todos los criterios para ser considerado un posible donante, será citado para acudir al centro autorizado una vez complete los 28 días de encontrarse asintomático. En el centro autorizado, el personal entrenado realizará:

- Entrevista clínica y evaluación médica siguiendo la norma vigente (R.M. Nº 241-2019/MINSA)

- Obtención del consentimiento para donación del estudio (Anexo 1) y del consentimiento y declaración jurada de acuerdo a normativa vigente de PRONAHEBAS.
- Extracción de alícuotas de sangre para el tamizaje inmunoserológico y calificación inmmunohematológica, según norma vigente
- Prueba serológica anti-SARS-CoV-2

Terminada la evaluación, se determinará si el paciente cumple con los criterios de la primera sección del check-list, en cuyo caso se encontrará apto para pasar a realizar la donación de plasma en el área designada para la colecta. El almacenamiento definitivo del plasma donado se realizará una vez se cuente con los resultados serológicos completos del donante, según norma vigente.

c) Colección del plasma
- La obtención de plasma se realizará por plasmaféresis
- Extraer un volumen máximo de plasma correspondiente al 15% de la volemia del donante. No sobrepasar la extracción de plasma de 600 cc.
- Plasma obtenido se dividirá antes de su congelación en 2-3 unidades, cada una de 200ml a 250ml
- Se separarán 5 alícuotas de 1.5 ml, las cuales deberán ser almacenadas a -80ºC

d) Procesamiento y manejo del plasma
- De encontrarse disponible, se realizará inactivación de patógenos con azul de metileno u otro procedimiento aprobado para este fin. De encontrarse disponible, se realizará la titulación de anticuerpos neutralizantes IgG anti-SARS-CoV-2, siguiendo las recomendaciones de PRONAHEBAS.
- El etiquetado de las unidades y de las alícuotas deberá contener un código único que permita la trazabilidad. En la etiqueta, se deberá indicar que la unidad es de uso exclusivo para manejo de pacientes con COVID-19 dentro de este ensayo clínico.
- El almacenamiento y transporte se realizará según normativa vigente

Todos los procedimientos mencionados se realizarán siguiendo las medidas de bioseguridad y el uso de equipo de protección personal (EPP) según la directiva vigente dictada por PRONAHEBAS.

6.2. Fase de uso monitoreado de plasma convaleciente:

El estudio se iniciará en la sede hospitalaria: Hospital Hipolito Unanue (Minsa), con los investigadores que forman parte de esta propuesta. Pudiendo ampliarse a otros centros de atención.

a) Selección de receptores y proceso de consentimiento informado

Los médicos tratantes de un paciente con diagnóstico de COVID-19 que consideren que podría ser candidato a recibir plasma convaleciente deberán:
- Completar el Check-list – Candidato a receptor a través de REDCap, verificando que el paciente cumpla con todos los criterios de inclusión y exclusión listados en la sección 5.2.
- Obtener el consentimiento informado (Anexo 2)

b) Proceso de solicitud de plasma y transporte
- Una vez que el Check-list - Candidato a receptor haya sido completado y el consentimiento informado haya sido subido al sistema informático del estudio, se contactará al banco de plasma centralizado para solicitar la liberación de 1 o 2 unidades de plasma convaleciente
- Se respetará la compatibilidad ABO del plasma entre el donante y el posible receptor.
- Se deberá mantener la trazabilidad entre donantes y receptores (registro específico)
- El transporte de la unidad de plasma deberá realizarse siguiendo la normativa vigente, manteniendo la cadena de frío.

c) Administración del plasma

El plasma convaleciente deberá transfundirse a los pacientes seleccionados utilizando procedimientos clínicos estándar de transfusión y únicamente después de obtener el consentimiento informado de su familiar directo.

- La dosis será 1 a 2 unidades de plasma de 200-250 ml
- La velocidad de infusión de una unidad se realizará de acuerdo a las condiciones clínicas del paciente, y no deberá ser mayor a 200 ml/hora
- El uso de medicinas pre-transfusión tales como paracetamol o difenhidramina se deja a criterio del médico tratante

d) Monitoreo durante la administración

Se deberá monitorizar al paciente durante la totalidad de la transfusión.

- Ante reacciones leves (picazón, quemazón en sitio de infusión) se deberá disminuir la velocidad de infusión o pausar la infusión. Si no se evidencia progresión de los síntomas se podrá continuar con la infusión a menor velocidad, según decisión del médico tratante.
- Ante reacciones alérgicas severas, se deberá descontinuar la infusión de inmediato, no ser reiniciada y brindar el manejo correspondiente.
    - Algunas de estas reacciones incluyen:
    - Manifestaciones mucosa o cutáneas como prurito intenso, eritema generalizado, edema de labios, lengua o úvula
    - Compromiso respiratorio: sibilantes, hipoxemia
    - Disminución de la presión arterial sistólica a < 90 mmHg o disminución >30% de la presión basal; o caída la presión diastólica en >30% de la presión basal
    - Taquicardia que incrementa a >130 latidos por minute; o bradicardia con <50 latidos por minuto.
    - Cualquier otro signo que en base al juicio del médico tratante requiere descontinuación de la transfusión.

Toda reacción observada deberá ser registrada en la historia clínica del paciente y en el CRF a través del sistema electrónico del estudio indicando el tiempo de aparición, manejo brindado, seguimiento e implicancia en el desarrollo del ensayo. Todo evento adverso serio deberá ser reportado según lo descrito en la sección 9.2 Definición de eventos adversos Serios y 9.3 Reporte de EAS
Cualquier tratamiento concomitante deberá ser registrado en el CRF electrónico. Esto incluye medicinas, suplementos vitamínicos o herbales, hemocomponentes y hemoderivados.

Los sujetos podrán recibir tratamiento concomitante para COVID-19 según el estándar utilizado en el centro de atención participante. Este tratamiento podrá incluir tocilizumab, hidroxicloroquina, azitromicina, remdesivir, Lopinavir/ritonavir, corticoides, entre otros. Sin embargo, los participantes no podrán participar de otros ensayos clínicos durante la totalidad de su participación en este estudio.

e) Evaluación inicial y seguimiento

El IP de cada sede hospitalaria supervisará la realización de las siguientes actividades y el registro de los siguientes datos en el sistema informático del estudio.

<u>Día -1 a 0:</u>

1. Tamizaje de receptor a través del llenado del check-list del receptor
2. Obtención de consentimiento informado
3. Enrolamiento (ingreso de datos de contacto y DNI en banco de identidades en REDCap)
4. Aleatorización
5. Evaluación basal del receptor (Información será obtenida de historia clínica y registrada en CRF)

- Información demográfica (edad, sexo)
- Fecha de inicio de síntomas
- Enfermedades crónicas concomitantes
- Medicación concomitante utilizada para COVID-19
- Resultado y fecha de prueba de laboratorio positiva para COVID-19
- Fecha de hospitalización
- Fecha de ingreso a UCI
- Fecha de intubación
- Nivel de requerimiento de oxigeno
- Hallazgos de TAC de pulmones más reciente (registrar fecha)

6. Evaluación laboratorial basal:
   - Prueba rápida serológica anti-SARS-CoV-2
   - Determinación de grupo sanguíneo, hemograma completo, perfil hepático (TGO, TGP, GGT, bilirubinas totales, albumina), creatinina, proteina C reactiva)
   - OPCIONAL: DHL, ferritina, dímero D
7. Registro de hora de inicio y fin de la infusión de plasma

<u>Día 0 hasta el alta hospitalaria o hasta el Día 30 (lo que ocurra primero)</u>

1. Fecha de fallecimiento
2. Reporte de eventos adversos serios y registro de eventos adversos
3. Actualización de medicación concomitante
4. Fecha de alta de UCI
5. Fecha de alta hospitalaria
6. Fecha de extubación
7. Fecha de discontinuación de soporte con oxígeno
8. En los días 3, 7 y 30:
   - Nivel de requerimiento de FiO_2
   - hemograma completo, perfil hepático (TGO, TGP, GGT, bilirubinas), creatinina
   - OPCIONAL: proteina C reactiva, DHL, ferritina, dímero D

6.3 Presentación, estabilidad, almacenamiento y transporte del producto del estudio

a) Etiquetas

El etiquetado de las unidades de plasma se realizará según los requisitos establecidos por el banco de sangre donde se realiza la donación. Las etiquetas contendrán una declaración de uso en investigación y deberán permitir la identificación inequívoca del uso exclusivo de estas unidades para el tratamiento de pacientes con confirmación de COVID-19.

b) Almacenamiento, transporte y Estabilidad

Las unidades de plasma obtenidas deberán mantenerse almacenadas en el banco de sangre centralizado, siguiendo normativas vigentes. El transporte de las unidades se realizará de forma tal, que se garantice el mantenimiento de la cadena de frio. La conservación de las unidades de plasma una vez descongeladas será entre 2 y 6ºC, debiendo transfundirse dentro de las 24 horas de ser descongelado, para garantizar la integridad de los anticuerpos neutralizantes.

El personal del centro debe utilizar las precauciones universales, y eliminar las jeringas y los componentes descartables del dispositivo sin aguja de conformidad con las políticas y las prácticas del centro.

7. SEGURIDAD Y EVENTOS ADVERSOS

7.1. Definición de eventos adversos

Un evento adverso se define como cualquier acontecimiento médico desfavorable en un paciente

de una investigación clínica a quien se le administró el producto en investigación, que no necesariamente tiene una relación causal con este tratamiento. Por lo tanto, un evento adverso puede ser cualquier signo (incluso un hallazgo de laboratorio anormal), síntoma desfavorable y/o involuntario o enfermedad asociada temporalmente con el uso del producto en investigación, ya sea que esté o no relacionado con el producto.

A partir del enrolamiento, los Investigadores deben realizar una evaluación de los eventos adversos. Todos los EA serán objeto de seguimiento hasta que se hayan resuelto o se consideren estables durante el transcurso del estudio. Si el evento tiene una fecha de resolución, esta deberá registrarse en el formulario de eventos adversos, y ser notificado a la autoridad reguladora.

7.2. Definición de evento adverso serio

El término "evento adverso serio" (EAS) se define como "un evento adverso o reacción adversa, que en la opinión del investigador, podría resultar en alguno de los siguientes resultados: Muerte, hospitalización del paciente o prolongación de esta, incapacidad persistente o importante, alteración considerable de la capacidad de llevar a cabo las funciones normales de la vida".

Dada la alta mortalidad de esta condición, solo la muerte dentro de las primeras 24 horas post-transfusión será reportada como EAS. La muerte pasadas las 24 horas no será reportada como EAS, al ser considerada como un criterio de valoración de eficacia, excepto si claramente se establece una relación causal entre la administración del producto y la ocurrencia de la muerte pasadas las 24 horas (por ejemplo, el participante desarrolla anafilaxis y como consecuencia de esta anafilaxis fallece pasadas las 24 horas)

En el caso específico de este estudio, algunos EAS a los que se deberá estar atentos es al desarrollo de los siguientes EAS:

- Anafilaxis;
- Rápido deterioro de la función respiratoria o del estado clínico del paciente (TRALI o TACO)
- Trastorno de la coagulación
- Rápido deterioro dentro de las 24 horas post-infusión.

7.3. Reporte de eventos adversos serios

Se recopilarán y notificarán los EAS a partir de la firma del consentimiento a lo largo del periodo del tratamiento e incluyendo el periodo de seguimiento post tratamiento establecido en el protocolo (hasta el alta hospitalaria o hasta cumplir los 30 días de seguimiento, ver sección 6.2). Adicionalmente los EAS producidos una vez finalizados los periodos antes señalados deberán ser notificados al patrocinador si el investigador toma conocimiento de éstos y considera que el EAS se relaciona con el producto de investigación.

El equipo de investigación deberá notificar al investigador principal la ocurrencia de cualquier evento adverso serio inmediatamente después de que se tome conocimiento de este evento y en un máximo de 6 horas. Esta notificación deberá quedar registrada en el CRF electrónico. Así mismo, deberá registrar todos los datos relacionados al EAS en la historia clínica del paciente y en el CRF electrónico.

El investigador deberá notificar los eventos adversos serios, las reacciones adversas serias y las sospechas de reacciones adversas serias e inesperadas al CNTEI, cuando suceda el hecho o tome conocimiento en un plazo **no mayor de un (1) día** calendario. Así mismo, deberá reportar a la OGITT del INS en un **plazo máximo de siete (7) días** calendario, a partir de sucedido el hecho o en cuanto tome conocimiento del hecho, a través del Sistema de Reporte Virtual de Eventos Adversos Serios (REAS-NET). La notificación inicial irá seguida de informes escritos pormenorizados brindando la información complementaria que se le soliciten. Por último, se remitirá un informe anual de seguridad a la OGITT.

8. CONSIDERACIONES ESTADISTICAS

8.1. Muestra

Utilizando la fórmula de Fleiss con corrección de continuidad y considerando una reducción de mortalidad de 50% a 20%, se requieren 45 pacientes por brazo. Adicionalmente, considerando un 10% de pérdida después del enrolamiento, planeamos enrolar un total de 100 pacientes, 50 por cada brazo.

Racionalidad:

- El Hospital Hipolito Unanue ha reportado una tasa de mortalidad de 50% en pacientes hospitalizados (Informe institucional).
- A la fecha, no existen publicados estudios clínicos aleatorizados que hayan evaluado la eficacia del plasma convaleciente en el manejo de COVID-19. Sin embargo, un reciente estudio de una cohorte de 5000 pacientes que recibieron plasma convaleciente para el manejo de COVID-19 severo o crítico, encontró que la mortalidad a 7 días fue de 14.9%. En este estudio también se reportó que la mortalidad de pacientes tratados previamente al estudio en los mismos hospitales fue de 21-50% (16).
- Realizando un cálculo conservador, se plantea buscar una reducción de mortalidad de 50% a 20%.

8.2. Análisis y procesamiento de los datos

Se presentarán las medidas de tendencia central y dispersión de las variables numéricas. Las variables categóricas se presentarán como frecuencias y porcentajes.

La proporción de eventos adversos, mortalidad y recuperación se realizará mediante ajuste exacto de intervalos de confianza por el método de Clopper-Pearson, así como por prueba de Kaplan-Meier y log rank-test.

Para las comparaciones entre los grupos, se utilizará la prueba exacta de Fisher o la prueba de comparación de proporciones de riesgos ajustada por Miettinen-Nurminen. Para las variables cuantitativas, se utilizará la prueba no paramétrica de Mann-Whitney.

8.3. Variables

Características del plasma

- Edad
- Fecha de primera prueba molecular positiva
- Fecha de primera prueba serológica
- Resultado de prueba serológica
- Fecha de última prueba molecular negativa
- Fecha de última prueba serológica
- Resultado de última prueba serológica
- Severidad de enfermedad superada
- Tiempo asintomático
- Título de anticuerpos neutralizantes (cuando se logren medir)
- Grupo sanguíneo

Características basales del receptor

- Pre-aleatorización: Edad, gestación, score SOFA
- Grupo sanguíneo
- Tiempo desde inicio de síntomas
- Tipo de confirmación de diagnóstico de COVID-19
- Severidad de enfermedad
- Nivel de requerimiento de oxigeno al momento de administración de plasma

- Tiempo en ventilación mecánica al momento de administración de plasma
- Laboratoriales: Presencia de IgG y/o IgM por prueba rápida serológica anti-SARS-CoV-2, resultados de hemograma, perfil hepático, creatinina, proteina C reactiva,
- Status serológico para VIH, Hepatitis B y Hepatitis C
- OPCIONAL: marcadores inflamatorios (DHL, ferritina, dímero D)

Para evaluación de eficacia

- Condición al alta
- Tratamiento concomitante para COVID-19 (con fecha de inicio y final)
- Número de días en ventilación mecánica
- Número de días en UCI
- Número de días de hospitalización
- Nivel de requerimiento de oxigeno en el día 3 y día 7
- OPCIONAL: marcadores inflamatorios a los 3 y 7 días (hemograma completo, perfil hepático (TGO, TGP, GGT, bilirrubinas totales, albumina), creatinina, DHL, proteína C reactiva, ferritina, dímero D)

Para evaluación de seguridad

- Muerte dentro de las 24 horas
- Evento adverso serio durante hospitalización
- Evento adverso durante hospitalización
- Resultados de hemograma completo, perfil hepático (TGO, TGP, GGT, bilirubinas), creatinina

9. RECOLECCIÓN DE DATOS Y MONITOREO DEL ENSAYO CLÍNICO

9.1. Ingreso de datos

- Se utilizará un código único de estudio para la identificación de los sujetos receptores y los donantes
- Todos los datos requeridos serán registrados en el CRF electrónico, a través un sistema electrónico seguro de gestión de datos clínicos por Internet (REDCap).
- El IP del centro mantendrá una lista de las personas debidamente calificadas a quienes se les hayan delegado obligaciones del estudio, quienes contarán con credenciales para ingresar al CRF electrónico
- La base de datos final del estudio contendrá solo información de-identificada y mantendrán en computadoras protegidas mediante contraseña.
- Solamente los investigadores o sus delegados tendrán acceso a las muestras y datos, los cuales podrán transferirse a otras bases de datos aprobadas según sea necesario para análisis.

9.2. Monitoreo del protocolo

Los investigadores del centro y el coordinador del estudio realizarán labores constantes de monitoreo de datos y cumplimiento del protocolo, de conformidad con el siguiente plan de monitoreo del estudio:

- Antes de la activación de un centro el investigador principal del centro presentará la documentación requerida: autorización del director del centro; lista de personal del estudio detallando su rol y presentando su CV y certificado de buenas prácticas clínicas no mayor a 3 años. La persona designada para realizar el registro de datos en el CRF electrónico deberá completar un entrenamiento específico para este estudio.
- Los investigadores de cada centro deberán garantizar el ingreso de los datos requeridos en

el CRF electrónico (REDCap). Este sistema contará con reglas de validación para minimizar errores al momento del ingreso de datos.

- El coordinador del estudio o el investigador principal realizará la revisión del cumplimiento del proceso de consentimiento informado y del cumplimiento de los criterios de elegibilidad, inmediatamente después de obtenido el consentimiento informado y enrolamiento del sujeto y previamente a la aleatorización del sujeto.
- Semanalmente se realizará una revisión retrospectiva de los datos ingresados para monitorizar que toda la información requerida haya sido ingresada en el CRF electrónico.

9.3. Monitoreo de seguridad y de futilidad

Un comité externo conformado por especialistas en diversas áreas (medicina intensiva, enfermedades infecciosas, hemoterapia, neumología, bioestadística, entre otros) se reunirá a los 3 meses del inicio del proceso de enrolamiento de participantes o al alcanzar el enrolamiento del 50% de participantes del estudio, si esto ocurre antes de los 3 meses, con el fin de analizar y evaluar los resultados preliminares en base a los siguientes criterios:

- Evaluación de eventos adversos serios reportados
- Mortalidad a los 7 días
- Duración de ventilación mecánica

Al término de esta asamblea se elaborará un informe el cual será remitido a la OGITT dentro de los plazos estipulados.

10. CONSIDERACIONES ÉTICAS

Esta investigación se realizará en cumplimiento del protocolo y los requisitos de regulación pertinentes.

Los investigadores principales serán responsables de obtener la aprobación o renovación anual del Comité Nacional Transitorio de Ética en Investigación (CNTEI) y de las instituciones necesarias, mientras dure el protocolo.

El IP del centro será responsable de asegurar la notificación oportuna al CNTEI de los EAS que se produjeron en el centro.

10.1. Consentimiento informado

Antes de que un sujeto pueda participar en el protocolo, el IP o su delegado deberá asegurarse de obtener el consentimiento informado del sujeto o su apoderado después de una explicación adecuada del propósito y los métodos, beneficios esperados y posibles riesgos del protocolo.

El formulario de consentimiento informado debe ser firmado y fechado personalmente por el paciente o un familiar cercano y por la persona que llevó a cabo la explicación del consentimiento informado (Anexo 2).

Las muestras biológicas obtenidas de los donantes serán transferidas a la UPCH para su almacenamiento y posterior uso en futuros estudios. Esto será explicado durante el proceso de consentimiento informado, el cual deberá ser realizado antes de la toma de las muestras y de la colecta del plasma.

10.2. Confidencialidad del sujeto

Tanto los donantes como los sujetos receptores serán identificados con un código único de estudio. Las muestras de investigación del estudio almacenadas se etiquetarán utilizando el

mismo código de estudio. Toda información registrada será almacenada utilizando estos códigos de estudio

El banco de identidades que asocia datos identificadores de los sujetos con el código de estudio, será almacenado de manera separada y encriptada, a la que solo los IP tendrán acceso.

10.3. Riesgos y beneficios

Potenciales riesgos: Aunque la transfusión de plasma convaleciente parece ser bastante segura, como cualquier terapia transfusional existen riesgos inherentes a la misma. A fin de reducir el riesgo de TRALI (causado por anticuerpos anti-HLA) los donantes será únicamente varones. Para prevenir la sobrecarga cardiaca asociada a transfusión (TACO) se evitara el uso de plasma en pacientes con insuficiencia cardiaca manifiesta. Para minimizar el riesgo de hemólisis se utilizara sangre ABO compatible.

Potenciales beneficios: La tasa de mortalidad en pacientes con COVID-19 que requieren ventilación mecánica es muy alta. Si se logra un efecto benéfico se podría disminuir en gran medida la mortalidad asociada a esta condición. Así mismo podría disminuir el tiempo de ventilación mecánica y hospitalización. Plan de uso y almacenamiento de muestras biológicas

10.4. Uso de muestras y datos

Las muestras, y los datos obtenidos conforme a este protocolo se podrán usar para conducir evaluaciones de seguridad y de respuesta inmunitaria relacionadas con el protocolo, así como evaluaciones futuras de laboratorio exploratorias relacionadas con la infección por COVID19, evaluaciones de laboratorio exploratorias relacionadas con la investigación de la vacuna o la enfermedad infecciosa en general, y para la validación del ensayo de investigación.

10.5. Compensación

No se contemplará una compensación económica para los pacientes o los donantes.

Para los donantes se contemplará el pago del transporte desde su casa al centro de donación.

10.6. Medidas de prevención de contagio por COVID-19

Con el fin de reducir al mínimo la posibilidad de que un donante se encuentre aún infectado por el SARS-CoV-2, solo se citarán al banco de sangre centralizado aquellas personas recuperadas que hayan estado asintomáticas por 28 días o más. De esta manera, aseguraremos que los potenciales donantes que vayan al banco de sangre centralizado no sean contagiantes.

Así mismo, a estos donantes se les proporcionará transporte privado ida y vuelta desde su domicilio al banco de sangre para evitar que se expongan a personas infectadas durante la movilización.

En el caso en el que el sujeto no se encuentre en capacidad de consentir, el proceso del consentimiento informado podrá efectuarse por vía telefónica con el familiar directo del paciente. De esta forma, se evitará exponer al familiar al ambiente hospitalario.

10.7. Medidas para evitar la saturación del sistema de salud

Se plantea el uso de un banco de sangre centralizado, con el fin de minimizar la posibilidad de saturación de otros bancos de sangre. Dentro del Banco de Sangre del INSN- San Borja, se instalará el banco de sangre centralizado para el plasma convaleciente. Para este fin se designará un área y personal destinado específicamente para realizar la entrevista pre-donación y colecta del plasma convaleciente, sin interrumpir el flujo normal de trabajo del banco de sangre regular. un area y personal

El coordinador de campo del estudio apoyará a los investigadores principales de cada centro en la recolección de información requerida, con el fin de evitar que estas actividades del estudio sobrecarguen a los médicos tratantes que trabajan en los hospitales.

11. COMUNICACIÓN Y PUBLICACIÓN DE LOS RESULTADOS

Los resultados de este estudio difundidos en eventos científicos, incluyendo conversatorios y foros académicos, y serán enviados para publicación en revistas indizadas.

Si el estudio es detenido tempranamente por el Comité de evaluación de seguridad, se procederá a comunicar inmediatamente estos resultados a los sujetos participantes, al CNTEI, al Ministerio de Salud y a la comunidad médica.

12. ACCESO POST ESTUDIO

Dado que la dosis estándar de plasma es de 1 a 2 unidades, los participantes del estudio no recibirán dosis adicionales culminado el estudio.

Si al término del estudio se encuentra que el uso de plasma convaleciente es seguro y efectivo, los resultados de éste estudio serán presentados inmediatamente al Ministerio de Salud y a la comunidad médica. Los procedimientos del estudio para la administración del plasma se encontrarán disponibles de manera abierta y gratuita para que pueda ser replicado en otras instituciones y ser incluido en las guías de manejo y documentos técnicos del Ministerio de Salud.

13. REFERENCIAS

1. World Health Organization. Coronavirus Disease (COVID-19) outbreak situation.

2. Wu Z, McGoogan JM. Characteristics of and Important Lessons From the Coronavirus Disease 2019 (COVID-19) Outbreak in China: Summary of a Report of 72314 Cases From the Chinese Center for Disease Control and Prevention. JAMA. 2020. Epub 2020/02/25. doi: 10.1001/jama.2020.2648. PubMed PMID: 32091533.

3. Ministerio de Salud. Comunicado Oficial de Prensa Nº 67. Perú.

4. Sahr F, Ansumana R, Massaquoi TA, Idriss BR, Sesay FR, Lamin JM, Baker S, Nicol S, Conton B, Johnson W, Abiri OT, Kargbo O, Kamara P, Goba A, Russell JB, Gevao SM. Evaluation of convalescent whole blood for treating Ebola Virus Disease in Freetown, Sierra Leone. J Infect. 2017;74(3):302-9. Epub 2016/11/22. doi: 10.1016/j.jinf.2016.11.009. PubMed PMID: 27867062; PMCID: PMC7112610.

5. Hung IF, To KK, Lee CK, Lee KL, Chan K, Yan WW, Liu R, Watt CL, Chan WM, Lai KY, Koo CK, Buckley T, Chow FL, Wong KK, Chan HS, Ching CK, Tang BS, Lau CC, Li IW, Liu SH, Chan KH, Lin CK, Yuen KY. Convalescent plasma treatment reduced mortality in patients with severe pandemic influenza A (H1N1) 2009 virus infection. Clin Infect Dis. 2011;52(4):447-56. Epub 2011/01/21. doi: 10.1093/cid/ciq106. PubMed PMID: 21248066.

6. Wong VW, Dai D, Wu AK, Sung JJ. Treatment of severe acute respiratory syndrome with convalescent plasma. Hong Kong Med J. 2003;9(3):199-201. Epub 2003/06/05. PubMed PMID: 12777656.

7. Cheng Y, Wong R, Soo YO, Wong WS, Lee CK, Ng MH, Chan P, Wong KC, Leung CB, Cheng G. Use of convalescent plasma therapy in SARS patients in Hong Kong. Eur J Clin Microbiol Infect Dis. 2005;24(1):44-6. Epub 2004/12/24. doi: 10.1007/s10096-004-1271-9. PubMed PMID: 15616839; PMCID: PMC7088355.

8. Ko JH, Seok H, Cho SY, Ha YE, Baek JY, Kim SH, Kim YJ, Park JK, Chung CR, Kang ES, Cho D, Muller MA, Drosten C, Kang CI, Chung DR, Song JH, Peck KR. Challenges of convalescent plasma infusion therapy in Middle East respiratory coronavirus infection: a single centre experience. Antivir Ther. 2018;23(7):617-22. Epub 2018/06/21. doi: 10.3851/IMP3243. PubMed PMID: 29923831.

9. Mair-Jenkins J, Saavedra-Campos M, Baillie JK, Cleary P, Khaw FM, Lim WS, Makki S, Rooney KD, Nguyen-Van-Tam JS, Beck CR, Convalescent Plasma Study G. The effectiveness of convalescent plasma and hyperimmune immunoglobulin for the treatment of severe acute respiratory infections of viral etiology: a systematic review and exploratory meta-analysis. J Infect Dis. 2015;211(1):80-90. Epub 2014/07/18. doi: 10.1093/infdis/jiu396. PubMed PMID: 25030060; PMCID: PMC4264590.

10. Casadevall A, Scharff MD. Return to the past: the case for antibody-based therapies in infectious diseases. Clin Infect Dis. 1995;21(1):150-61. Epub 1995/07/01. doi: 10.1093/clinids/21.1.150. PubMed PMID: 7578724; PMCID: PMC7197598.

11. World Health Organization. Blood Regulators Network Position Paper on Use of Convalescent Plasma, Serum or Immune Globulin Concentrates as an Element in Response to an Emerging Virus (2017). .

12. World Health Organization. Ethical issues related to study design for trials on therapeutics for Ebola Virus Disease. 2014. Report of the WHO Ethics Working Group meeting, 20–21 October 2014.

13. US Federal Drug Administration (FDA). Recommendations for Investigational COVID-19 Convalescent Plasma. April 8, 2020, EE.UU.

14. Organización Mundial de la Salud. Mantenimiento de un suministro seguro y suficiente de sangre durante los brotes de enfermedad por coronavirus 2019 (COVID-19): Orientaciones provisionales (20 de marzo 2020).

15. Johns Hopkins. Convalescent Plasma to Treat Coronavirus - Associated Severe Pulmonary Complications: A Feasibility Study Assessing the Safety of Multiple Doses of Anti-SARS-CoV-2 Plasma in Mechanically Ventilated Intubated Patients with Respiratory Failure due to COVID-19 (CPPulm-001), Clinical Phase: 1.

16. Joyner M. Expanded Access to Convalescent Plasma for the Treatment of Patients with COVID-19. Version 2.0, 03 April 2020. Unique Protocol Identification Number: 20-003312 National Clinical Trial (NCT) Identified. Funded by: BARDA. EE.UU.

17. Ministerio de Sanidad de España. Recomendaciones para la obteciónobtención de plasma de donantes convalecientes de COVID-19. Comité Científico para la Seguridad Transfusional (CCST). Versión 1.0 – 26 marzo de 2020. .

18. Richardson S, Hirsch JS, Narasimhan M, Crawford JM, McGinn T, Davidson KW, and the Northwell C-RC, Barnaby DP, Becker LB, Chelico JD, Cohen SL, Cookingham J, Coppa K, Diefenbach MA, Dominello AJ, Duer-Hefele J, Falzon L, Gitlin J, Hajizadeh N, Harvin TG, Hirschwerk DA, Kim EJ, Kozel ZM, Marrast LM, Mogavero JN, Osorio GA, Qiu M, Zanos TP. Presenting Characteristics, Comorbidities, and Outcomes Among 5700 Patients Hospitalized With COVID-19 in the New York City Area. JAMA. 2020. Epub 2020/04/23. doi: 10.1001/jama.2020.6775. PubMed PMID: 32320003; PMCID: PMC7177629.

19. Wang K, Zhang Z, Yu M, Tao Y, Xie M. 15-day mortality and associated risk factors for hospitalized patients with COVID-19 in Wuhan, China: an ambispective observational cohort study. Intensive Care Med. 2020. Epub 2020/04/25. doi: 10.1007/s00134-020-06047-w. PubMed PMID: 32328724; PMCID: PMC7176814.

20. Yang X, Yu Y, Xu J, Shu H, Xia J, Liu H, Wu Y, Zhang L, Yu Z, Fang M, Yu T, Wang Y, Pan S, Zou X, Yuan S, Shang Y. Clinical course and outcomes of critically ill patients with SARS-CoV-2 pneumonia in Wuhan, China: a single-centered, retrospective, observational study. Lancet Respir Med. 2020;8(5):475-81. Epub 2020/02/28. doi: 10.1016/S2213-2600(20)30079-5. PubMed PMID: 32105632; PMCID: PMC7102538.

21. Cao WC, Liu W, Zhang PH, Zhang F, Richardus JH. Disappearance of antibodies to SARS-associated coronavirus after recovery. N Engl J Med. 2007;357(11):1162-3. Epub 2007/09/15. doi: 10.1056/NEJMc070348. PubMed PMID: 17855683.

22. To KK, Tsang OT, Leung WS, Tam AR, Wu TC, Lung DC, Yip CC, Cai JP, Chan JM, Chik TS, Lau DP, Choi CY, Chen LL, Chan WM, Chan KH, Ip JD, Ng AC, Poon RW, Luo CT, Cheng VC, Chan JF, Hung IF, Chen Z, Chen H, Yuen KY. Temporal profiles of viral load in posterior oropharyngeal saliva samples and serum antibody responses during infection by SARS-CoV-2: an observational cohort study. Lancet Infect Dis. 2020;20(5):565-74. Epub 2020/03/28. doi: 10.1016/S1473-3099(20)30196-1. PubMed PMID: 32213337; PMCID: PMC7158907.

23. Duan K, Liu B, Li C, Zhang H, Yu T, Qu J, Zhou M, Chen L, Meng S, Hu Y, Peng C, Yuan M, Huang J, Wang Z, Yu J, Gao X, Wang D, Yu X, Li L, Zhang J, Wu X, Li B, Xu Y, Chen W, Peng Y, Hu Y, Lin L, Liu X, Huang S, Zhou Z, Zhang L, Wang Y, Zhang Z, Deng K, Xia Z, Gong Q, Zhang W, Zheng X, Liu Y, Yang H, Zhou D, Yu D, Hou J, Shi Z, Chen S, Chen Z, Zhang X, Yang X. Effectiveness of convalescent plasma therapy in severe COVID-19 patients. Proc Natl Acad Sci U S A. 2020;117(17):9490-6. Epub 2020/04/08. doi: 10.1073/pnas.2004168117. PubMed PMID: 32253318; PMCID: PMC7196837.

24. Wu F, Wang A, Liu M, Wang Q, Chen J, Xia S, Ling Y, Zhang Y, Xun J, Lu L, Jiang S, Lu H, Wen Y, Huang J. Neutralizing antibody responses to SARS-CoV-2 in a COVID-19 recovered patient cohort and their implications. medRxiv. 2020:2020.03.30.20047365. doi: 10.1101/2020.03.30.20047365.

25. Shen C, Wang Z, Zhao F, Yang Y, Li J, Yuan J, Wang F, Li D, Yang M, Xing L, Wei J, Xiao H, Yang Y, Qu J, Qing L, Chen L, Xu Z, Peng L, Li Y, Zheng H, Chen F, Huang K, Jiang Y, Liu D, Zhang Z, Liu Y, Liu L. Treatment of 5 Critically Ill Patients With COVID-19 With Convalescent Plasma. JAMA. 2020. Epub 2020/03/29. doi: 10.1001/jama.2020.4783. PubMed PMID: 32219428; PMCID: PMC7101507.

26. Ye M, Fu D, Ren Y, Wang F, Wang D, Zhang F, Xia X, Lv T. Treatment with convalescent plasma for COVID-19 patients in Wuhan, China. J Med Virol. 2020. Epub 2020/04/16. doi: 10.1002/jmv.25882. PubMed PMID: 32293713.

27. Ahn JY, Sohn Y, Lee SH, Cho Y, Hyun JH, Baek YJ, Jeong SJ, Kim JH, Ku NS, Yeom JS, Roh J, Ahn MY, Chin BS, Kim YS, Lee H, Yong D, Kim HO, Kim S, Choi JY. Use of Convalescent Plasma Therapy in Two COVID-19 Patients with Acute Respiratory Distress Syndrome in Korea. J Korean Med Sci. 2020;35(14):e149. Epub 2020/04/14. doi: 10.3346/jkms.2020.35.e149. PubMed PMID: 32281317; PMCID: PMC7152526.

28. Zhang B, Liu S, Tan T, Huang W, Dong Y, Chen L, Chen Q, Zhang L, Zhong Q, Zhang X, Zou Y, Zhang S. Treatment With Convalescent Plasma for Critically Ill Patients With SARS-CoV-2 Infection. Chest. 2020. Epub 2020/04/04. doi: 10.1016/j.chest.2020.03.039. PubMed PMID: 32243945; PMCID: PMC7195335.

29. Zeng QL, Yu ZJ, Gou JJ, Li GM, Ma SH, Zhang GF, Xu JH, Lin WB, Cui GL, Zhang MM, Li C, Wang ZS, Zhang ZH, Liu ZS. Effect of Convalescent Plasma Therapy on Viral Shedding and Survival in COVID-19 Patients. J Infect Dis. 2020. Epub 2020/04/30. doi: 10.1093/infdis/jiaa228. PubMed PMID: 32348485; PMCID: PMC7197534.

30. Wan Y, Shang J, Sun S, Tai W, Chen J, Geng Q, He L, Chen Y, Wu J, Shi Z, Zhou Y, Du L, Li F. Molecular Mechanism for Antibody-Dependent Enhancement of Coronavirus Entry. J Virol. 2020;94(5). Epub 2019/12/13. doi: 10.1128/JVI.02015-19. PubMed PMID: 31826992; PMCID: PMC7022351.
